# New insights into ginsenoside Rg1 regulating the niche to inhibit age-induced germline stem cells depletion through targeting ECR/BMP signaling pathway in *Drosophila*

**DOI:** 10.18632/aging.205548

**Published:** 2024-02-14

**Authors:** Baoyu Fu, Rui Ma, Fangbing Liu, Xuenan Chen, Manying Wang, Wenqi Jin, Shuai Zhang, Yanping Wang, Liwei Sun

**Affiliations:** 1 Research Center of Traditional Chinese Medicine, Affiliated Hospital, Changchun University of Chinese Medicine, Changchun, Jilin 130021, China; 2 Northeast Asia Institute of Traditional Chinese Medicine, Changchun University of Chinese Medicine, Changchun, Jilin 130117, China; 3 Obstetrics and Gynecology Diagnosis and Treatment Center, The Affiliated Hospital, Changchun University of Chinese Medicine, Changchun, Jilin 130062, China; 4 Key Laboratory of Active Substances and Biological Mechanisms of Ginseng Efficacy, Ministry of Education, Changchun University of Chinese Medicine, Changchun, Jilin 130117, China

**Keywords:** ginsenoside Rg1, ecdysterone receptor, niche, germline stem cells, BMP signaling

## Abstract

Purpose: The age-induced imbalance in ecological niches leads to the loss of GSCs, which is the main reason for ovarian germline senescence. Ginsenoside Rg1 can delay ovarian senescence. Here, we shed light on new insights of ginsenoside Rg1 in regulating the niche to maintain GSCs self-renewal and discussing related molecular mechanisms.

Methods: The differences among GSC number, reproductive capacity of naturally aging female *Drosophila* after ginsenoside Rg1 feeding were analyzed by immunofluorescence and behavior monitoring. The expressions of the active factors in the niche and the BMP signaling were analyzed through Western blot and RT-qPCR. The target effect was verified in the ECR mutant and combined with the molecular docking.

Results: Ginsenoside Rg1 inhibited the age-induced reduction of the GSCs number and restored offspring production and development. Ginsenoside Rg1 promoted the expression of anchor proteins E-cadherin, stemness maintenance factor Nos and differentiation promoting factor Bam, thereby GSCs niche homeostasis was regulated. In addition, ginsenoside Rg1 was bound to the LBD region of the hormone receptor ECR. Ginsenoside Rg1 promotes the regeneration of GSCs by targeting the ECR to increase pSmad1/5/8 expression and thereby activating the BMP signaling pathway. In addition, ginsenoside Rg1 maintenance of niche homeostasis to promote GSCs regeneration is dependent on ECR as demonstrated in ECR mutants.

Conclusions: Ginsenoside Rg1 regulated the ecological niche homeostasis of GSCs and promoted the regeneration of GSCs by targeting the ECR/BMP signaling pathway in hormone-deficient states in aging ovaries. It is of great significance for prolonging fertility potential and delaying ovarian senescence.

## INTRODUCTION

Stem cells have self-renewal ability and multi-directional differentiation potential. They also exhibit tissue repair capabilities and play a crucial role in maintaining tissue homeostasis [1]. Germline stem cells (GSCs) as the cornerstone of ovarian function, can continuously produce new oocytes to maintain ovarian reproductive and endocrine functions [2]. Studies have shown that the GSCs reduction directly determines the aging of the ovaries, causing women to face the symptoms of menopause and low estrogen levels [3]. GSCs can only multiply and maintain self-renewal in a specific niche. The niche of GSCs is the surrounding microenvironment, which controls the biological properties of GSCs in several ways, such as nutrient supply and cytokine secretion [4]. Some studies indicate that the reduced regeneration of the oocytes in the decaying ovary is a result of the disruption of the niche for GSCs [5]. The unhealthy niche causes the stem cell self-renewal to be impaired [6, 7], which ultimately leads to decreased oocyte generation, declining female fertility, and age-related diseases, such as osteoporosis and ovarian cancer. Other studies suggest that diet and exercise can improve the GSCs niche of ovarian aging, increase endogenous GSCs viability and improve ovarian reserve capacity [4, 8]. Therefore, healthy GSCs niche ensures the continued regeneration of GSCs, which is beneficial for women’s health and life.

The GSCs niche is affected by a variety of signals [9]. In this niche, bone morphology protein (BMP) signaling is a key signal that regulates GSCs regeneration [10]. BMP signaling plays an important role in regulating early embryonic development, ovarian granulosa cell proliferation, oocyte maturation and reproductive hormone synthesis, depending on the ovarian paracrine/autocrine regulatory systems [11]. Several studies have demonstrated that activation of BMP signaling in the GSCs niche can promote the activity of stem cell maintenance factor Nos and differentiation factor Bam [12, 13]. In addition, the crosstalk between the GSCs and their niche depends on the cell adhesion, whereas E-cadherin plays an important role in this adhesion junction. GSCs, with a high expression of E-cadherin, are much more competitive than neighboring cells in forming the stem cell niche [14]. The BMP signals affect the transcription levels of Nos, Bam and E-cadherin [15]. The niche in the aging ovary was weakened, resulting in a reduction in the number of GSCs [16]. Therefore, BMP signals can maintain GSCs niche homeostasis and determine GSCs regeneration. Ginsenosides are important pharmacological components of *Panax* ginseng with anti-aging effects [17]. Studies have shown that ginsenosides can promote stem cell regeneration, regulate endocrine system and prevent follicular atresia [18, 19]. Our previous studies have shown that total ginsenosides could activate steroid signals to improve the reproductive capacity of aged female *Drosophila* [20]. Ginsenosides Rg1 has numerous pharmacological effects in antiovarian aging. He et al. believed that ginsenoside Rg1 might improve the fertility of D-gal-induced aging mice and reduce the pathological damage of ovary by enhancing the anti-inflammation, anti-oxidation and reducing the expression related to aging proteins [21]. Similarly, ginsenoside Rg1 exhibited the ability to mitigate the pathological deterioration of the ovary and uterus in an aging mouse model through its antioxidative and anti-inflammatory properties. Furthermore, it demonstrated the potential to enhance the reproductive function of aging mice affected by D-galactose-induced impairment by up-regulating the expression of granulocyte follicle-stimulating hormone receptor and down-regulating the aging signaling pathways P53-P21-P19/stk, P16, and Bax/Bcl-2 [22]. Dai et al. showed that long-term administration of ginsenoside Rg1 could improve the fertility of naturally aging mice, which might be related to the obvious antioxidant effect on the ovary [23, 24]. The antiovarian aging activities of Rg1 have received extensive attention, but it inhibited age-induced GSCs loss has not been clarified.

In this study, we aimed to evaluate ginsenoside Rg1 maintaining GSCs niche homeostasis through BMP signaling pathways in a naturally aged female *Drosophila* model. These findings could provide new insights into the molecular mechanism of ginsenoside Rg1 acts as an anti-aging agent for the ovaries.

## MATERIALS AND METHODS

### *Drosophila* maintenance and grouping

**Figure 1:**
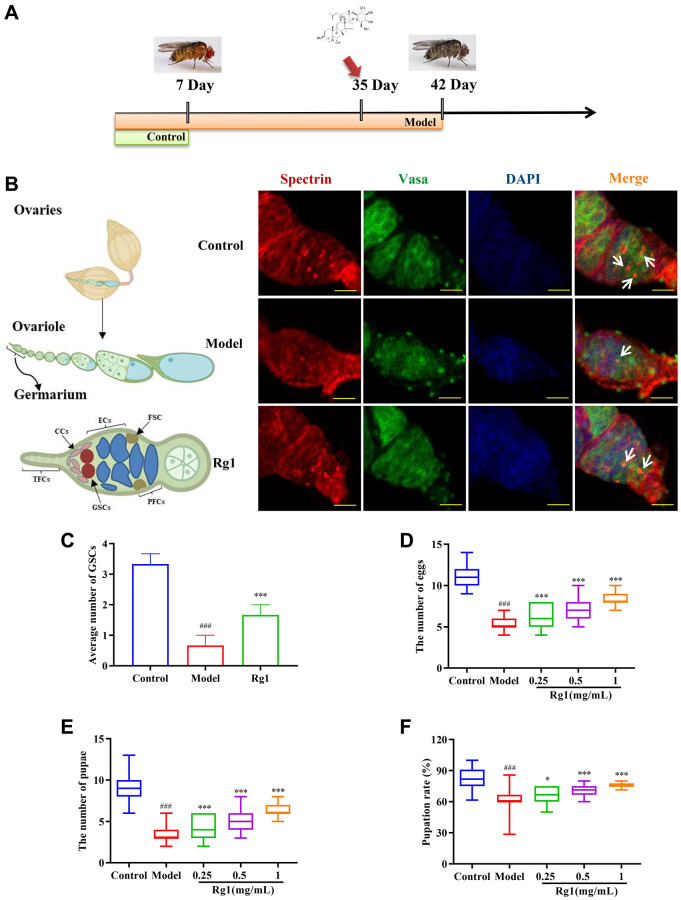
**Effects of ginsenoside Rg1 on GSCs regeneration.** (**A**) Design procedures for animal experiment; (**B**) GSCs are labeled with α-spectrin antibody (red, fusions), Vasa antibody (green, germ cells), and DAPI (blue, nuclei), Scale bar: 10 μm; (**C**) Average number of GSCs; (**D**) Number of eggs (*n* = 100); (**E**) Number of pupae (*n* = 100); (**F**) Pupation rate. Results were analyzed with one-way ANOVA. Data are shown as the mean ± SD; ^###^*p* < 0.001 compared with Control (7-day-old female *Drosophila*); ^*^p < 0.05, ^***^p < 0.001 compared with Model (35-day-old female *Drosophila*).

*Drosophila* were acquired from the Experimental Center, the Affiliated Hospital of the Changchun University of Chinese Medicine’s *Drosophila* Research Repository. (Changchun, China). ECR mutant *Drosophila* (Shanghai, China) were provided by the Core Facility of *Drosophila* Resource and Technology, CEMCS, CAS. In order to cultivate *Drosophila,* a cornmeal-sugar-yeast agar standard complete nutrient medium was used, which contained 8% sugar, 10.8% cornmeal, 0.5% propionic acid, 0.5% Oxoid LP0021 yeast extract (all from Maikelin, Shanghai, China), and 2% agar (Biofroxx, Guangzhou, China). All *Drosophila* were kept and raised at a temperature of 25°C and a humidity of 60% with a light-dark cycle of 12:12 hours. According to the procedure presented in Figure 1A, five groups of virgin *Drosophila*, including the model group (42-day old female *Drosophila*), three ginsenoside Rg1 treatment groups (35-day old female *Drosophila*), and the control group (7-day young female *Drosophila*), were randomly assigned. The ginsenoside Rg1 treatment groups were supplemented with ginsenoside Rg1 (Yuanye Bio-Technology Co., Ltd., Shanghai, China) at final concentrations of 0.25, 0.5, and 1 mg/mL in the culture medium and fed for 7 days. Subsequent experiments followed this grouping.

### Reproduction assays

The control and model groups were fed with standard basal medium, and the remaining three treatment groups were fed with 0.25, 0.5, and 1 mg/mL of ginsenoside Rg1 in basal medium. After 7 days, the flies in each group were transferred to new tubes containing basal medium to lay eggs for 48 h. The eggs and pupae were counted, and the pupation rate per tube was computed, in order to assess the impacts of ginsenoside Rg1 on the growth and reproduction of female *Drosophila* [25]. Results for assessments derived from three separate trials were provided as mean ± standard deviation (SD).

### Immunofluorescence staining

A small incision was made at the very back of the female *Drosophila* abdomen using forceps. Then, the ovaries were extruded from the abdomen from front to back and placed in a dissecting buffer. Finally, the adult ovaries were removed by stripping as the fat, fibers, and other tissues of the abdomen, which might have been brought out from around the ovaries. The stripped ovaries were preserved for 20 min at RT with 4% paraformaldehyde, followed by three washings with PBST (0.1% Triton X-100 in 1 × PBS). The primary antibodies, mouse anti-α-spectrin (1:20, DSHB, NIH, USA) and rat polyclonal anti-Vasa (1:50, DSHB, NIH, USA), dissolved in blocking solution (with 1% normal goat serum (Solarbio, Beijing, China) in 0.1% PBST) overnight at 4°C, were then treated with the blocked ovaries for at least 20 min at RT. The ovaries were washed three times, blocked for 20 min, and then incubated at RT for two hours with secondary antibodies Alexa Fluor 488-conjugated goat anti-rat, Alexa Fluor 647-conjugated goat anti-mouse, and DAPI (1:1,000, Sigma-Aldrich, Nasdaq, USA). A micro slicing tool was used for manipulating the ovaries in 1 × PBS for ovariole individualization, and the ovary was mounted in antifade mounting medium (Solarbio, Beijing, China). A Leica SP8 confocal lens (Wetzlar, Germany) was used for capturing the images [26].

### Reverse transcription-quantitative PCR (RT-qPCR)

The total RNA was obtained by a Magnetic Tissue/Cell/Blood Total RNA Kit (Invitrogen, USA). 100 stripped ovaries from each group were added 450 μL lysis solution and 5 magnetic beads, and then put into a tissue crusher at 4°C, 12000 r/min for 5 min. The supernatant was extracted. Using the script cDNA synthesis reagent (Bio-Rad, USA), 1 μg of RNA was reverse-transcribed into cDNA. Then, RT-qPCR was carried out in a Bio-Rad CFX96 instrument using the SYBR Premix Ex Taq Kit (Takara Biomedical Technology Co., Ltd., Beijing, China) under the following conditions for 40 cycles: 95°C for 5 min, 95°C for 15 seconds, 60°C for 30 seconds, and 72°C for 30 seconds. The β-actin gene was the internal control. The ratios between the values in each group and the equivalent values received from the control group were used to determine the amounts of gene expression using the threshold cycle (Ct) technique. The levels were compared among groups. Primers used for RT-qPCR included: forward, 5′-GCT CTG GAG TTG GAC AAT GGC TAT C -3′ and reverse, 5′-ATC TTG ATG TGC TGA TGG CGG ATG -3′ for E-cadherin; 5′-CGC TGG ACT CGC ACG ACT ATT G -3′ and reverse, 5′-CGC TCT GCT GCT GCT GAC TTA G -3′ for ECR; and forward, 5′-TTG TCT GGG CAA GAG GAT CAG -3′ and reverse, 5′-ACC ACT CGC ACT TGC ACT TTC -3′ for β-actin [27].

### Western blot analysis

The total protein of the ovary was obtained, by utilizing lysate buffer (Beyotime, Haimen, China) and Bullet Blender (Nikon, Tokyo, Japan). Ovarian tissues were placed in RIPA lysis solution and broken at 4°C using an ultrasonic crusher. The supernatant was centrifuged and collected after being placed on ice for 40 min. Protein concentration was assessed using BCA Protein Assay Kit. GAPDH (1:50000, BM1623; Boster, Beijing, China) was utilized as the loading control. Protein samples had been separated on 12% SDS-PAGE (Bio-Rad, USA). After blocked with 5% non-fat milk, PVD membranes were immunoblotted with the following antibodies: ISWI (1:1000, ab 195309), Nos (2 μg/ml, ab 70001) (both from Abcam, USA); Smad1/5/8 (#12656), pSmad1/5/8 (#13820) (1:500, both from Cell Signaling Technology, USA); Decapentaplegic (Dpp) (1:100, sc-133182), Smad4 (1:200, sc-7966) (both from Santa Cruz Biotechnology, USA), and Bam (3 μg/ml, DSHB, NIH, USA). The membrane was treated with the proper secondary antibodies (Li-Cor, USA) at RT for 1 hour [28]. Subsequently, protein bands were visualized and evaluated via chemiluminescence using a FluorChem imaging device (ProteinSimple, USA).

### Molecular docking of ecdysterone and ginsenoside Rg1 with ECR

The binding mode and interaction of ecdysterone and ginsenoside Rg1 with ECR_DROME protein were evaluated. The binding pocket was identified by blind docking. In order to predict the binding site of the protein, a 40Å length cubic box was generated while set at the center for the predicted binding sites. This box was represented by a set of evenly distributed grid points with a grid space of 2Å [29]. Exhaustibility was assigned to set at 8. Additional limitations were predetermined as defaults for Autodock Vina and are not discussed here [30]. The highest binding affinities of ecdysterone and ginsenoside Rg1 were visibly investigated using Pymol and the Discovery Studio Visualizer programs.

### Statistical analysis

Each trial included a minimum of three biological replicas. The data were presented as mean ± SD. Data were normally distributed and Shapiro-Wilk test and D’Agostino-Pearson omnibus K2 test were used to determine normality. All data with a normal distribution and consistent deviations were examined for normalcy and homogeneity before being subjected to one-way ANOVA analysis. GraphPad Prism 8 was used to conduct the statistical analysis (GraphPad Software, USA). One-way ANOVA multiple comparison tests (Tukey’s post hoc) were used to ascertain the findings’ statistical significance; a value of *p* < 0.05 was deemed statistically significant.

## RESULTS

### Ginsenoside Rg1 promoted GSCs regeneration

The failure of ovarian function was associated with the depletion of stem cell populations. We have observed that the average number of GSCs in older *Drosophila* was 0.67 and that in younger *Drosophila* was 3.33. The treatment of ginsenoside Rg1 increased the average number of GSCs in the aging ovary to 1.67, inhibiting the depletion of stem cells (Figure 1B). Ovarian function attenuation leads to the degradation of reproductive capacity and the reduction of the quantity and quality of offspring [31]. In comparison to young *Drosophila* (the control group), the quantity of eggs and pupae as well as the pupation rate of aged *Drosophila* (the model group) were considerably lower, indicating that their reproductive ability had been compromised. Ginsenoside Rg1 substantially and concentration-dependently increased the number of eggs, pupae, and pupation rate in elderly female *Drosophila* (Figure 1C–1F). In older *Drosophila* treated with 1 mg/mL ginsenoside Rg1, the quantity of eggs increased by 49.63%, the quantity of pupae increased by 75.21%, and the pupation rate increased by 17.72% respectively.

The typical manifestations of decreased ovarian function are female hormones deficiency and morphological atrophy. Ecdysterone is a steroid hormone secreted by the ovaries, similar to female hormones, that regulates follicular development and induces oocyte maturation in female *Drosophila.* Ecdysterone level in aged female *Drosophila* was increased by ginsenoside Rg1 in a dose-dependent manner (Supplementary Figure 1A). Ovarian atrophy was alleviated in old female *Drosophila* after treatment with 1 mg/mL ginsenoside Rg1 (Supplementary Figure 1B–1D). In addition, reduced ovarian function accelerated overall aging and degeneration, resulting in a shorter lifespan and reduced motor activity. While ginsenoside Rg1 enhanced ovarian function, it also restored overall youth in aging female *Drosophila* (Supplementary Figure 2).

### Ginsenoside Rg1 regulated the homeostasis of GSCs niche

**Figure 2:**
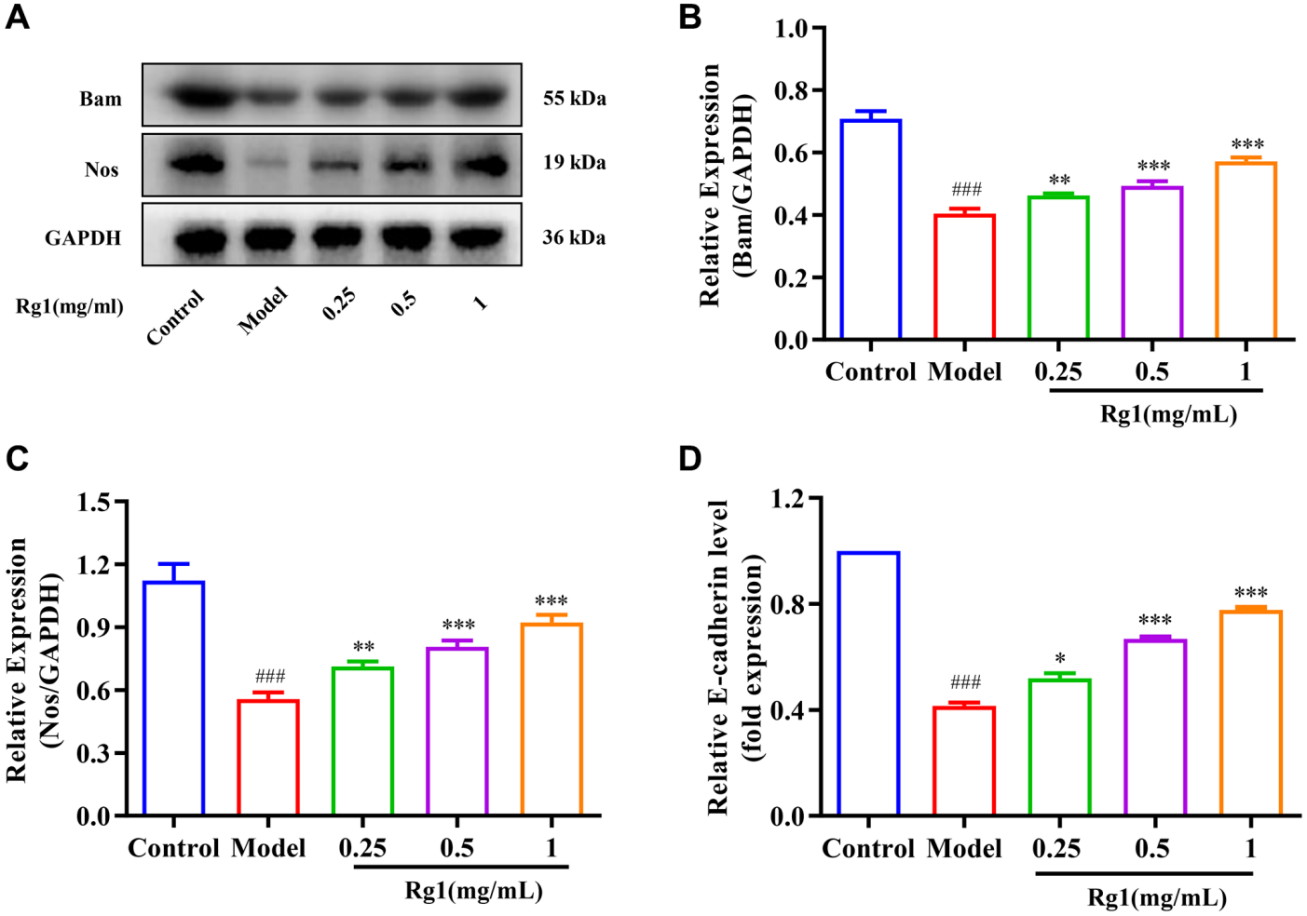
**Effect of ginsenoside Rg1 on the niche of GSCs.** (**A**) Western blotting analysis of expression of Bam and Nos; (**B**) Relative expression levels of Bam; (**C**) Relative expression levels of Nos; (**D**) Relative mRNA-level expression of gene E-cadherin; GAPDH antibody was used as loading Control. Results were analysed with one-way ANOVA. Data are shown as the mean ± SD (*n* = 100); ^###^*p* < 0.001 compared with Control (7-day-old female *Drosophila*); ^*^p < 0.05, ^**^p < 0.01, ^***^p < 0.001 compared with Model (35-day-old female *Drosophila*).

The regeneration of GSCs is directly regulated by their niche. The ovarian niche transmits important cytokines to GSCs. For example, Nos and Bam regulate the stemness and differentiation of reproductive stem cells. E-cadherin in the niche maintains the stem cell population by immobilizing GSCs to prevent shedding. The expression of Nos, Bam and E-cadherin decreased significantly in aged *Drosophila*, resulting in niche imbalance (Figure 2). Ginsenoside Rg1 substantially increased the Bam, Nos, and E-cadherin expression in the aging GSCs niche (Figure 2A–2D). According to these findings, ginsenoside Rg1 can inhibit age-induced niche imbalance.

### Ginsenoside Rg1 activates BMP signaling pathway

**Figure 3:**
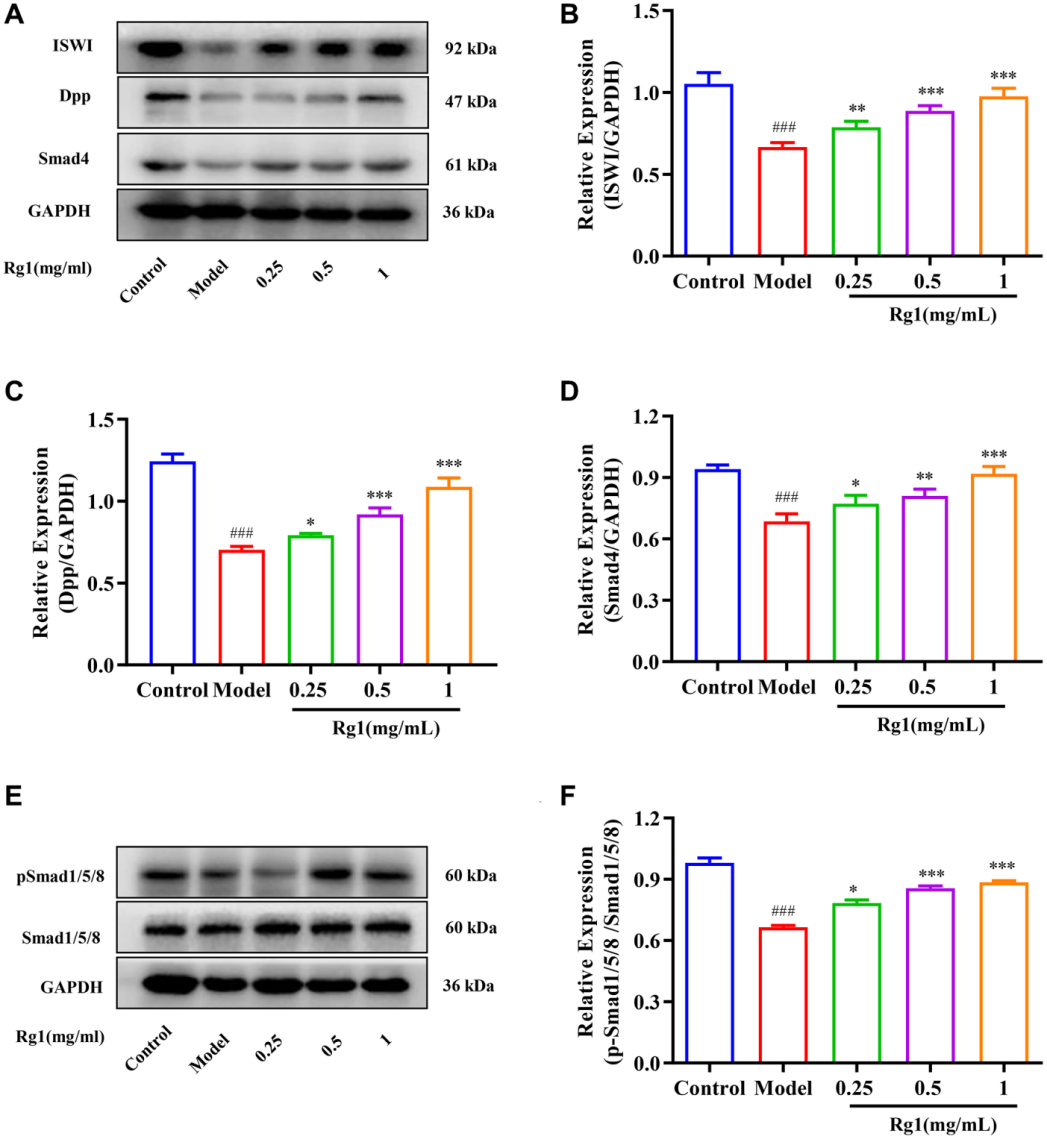
**Effect of ginsenoside Rg1 on niche BMP signaling pathway.** (**A**) Western blotting analysis of expression of ISWI, Dpp and Smad 4; (**B**) Relative expression levels of ISWI; (**C**) Relative expression levels of Dpp; (**D**) Relative expression levels of Smad4; (**E**) Western blotting analysis of expression of pSmad1/5/8; (**F**) Relative expression levels of pSmad1/5/8; GAPDH antibody was used as loading Control. Results were analysed with one-way ANOVA. Data are shown as the mean ± SD (*n* = 100); ^###^*p* < 0.001 compared with Control (7-day-old female *Drosophila*); ^*^p < 0.05, ^**^p < 0.01, ^***^p < 0.001 compared with Model (35-day-old female *Drosophila*).

The BMP signal is the most important signal to regulate niche activity. Diminished BMP signaling in niche leads to decreased GSCs and reduced ovarian function consequently. When exposed to higher concentration of ginsenoside Rg1, the expression of ISWI and the BMP ligand Dpp in the ovaries of aged *Drosophila* was promoted (Figure 3A–3C). In addition, the molecular marker for BMP signal activation is phosphorylation Smad1/5/8, which forms a complex with Smad4 to control Nos and Bam expression in GSCs niche. The expression of pSmad1/5/8 and Smad4 were significantly increased in aged female *Drosophila* with ginsenoside Rg1 treatment (Figure 3D–3F). These suggested that ginsenoside Rg1 activates BMP signaling to regulate the homeostatin of GSCs niche, and promote GSCs regeneration.

### Ginsenoside Rg1 increased ECR expression and activity

**Figure 4:**
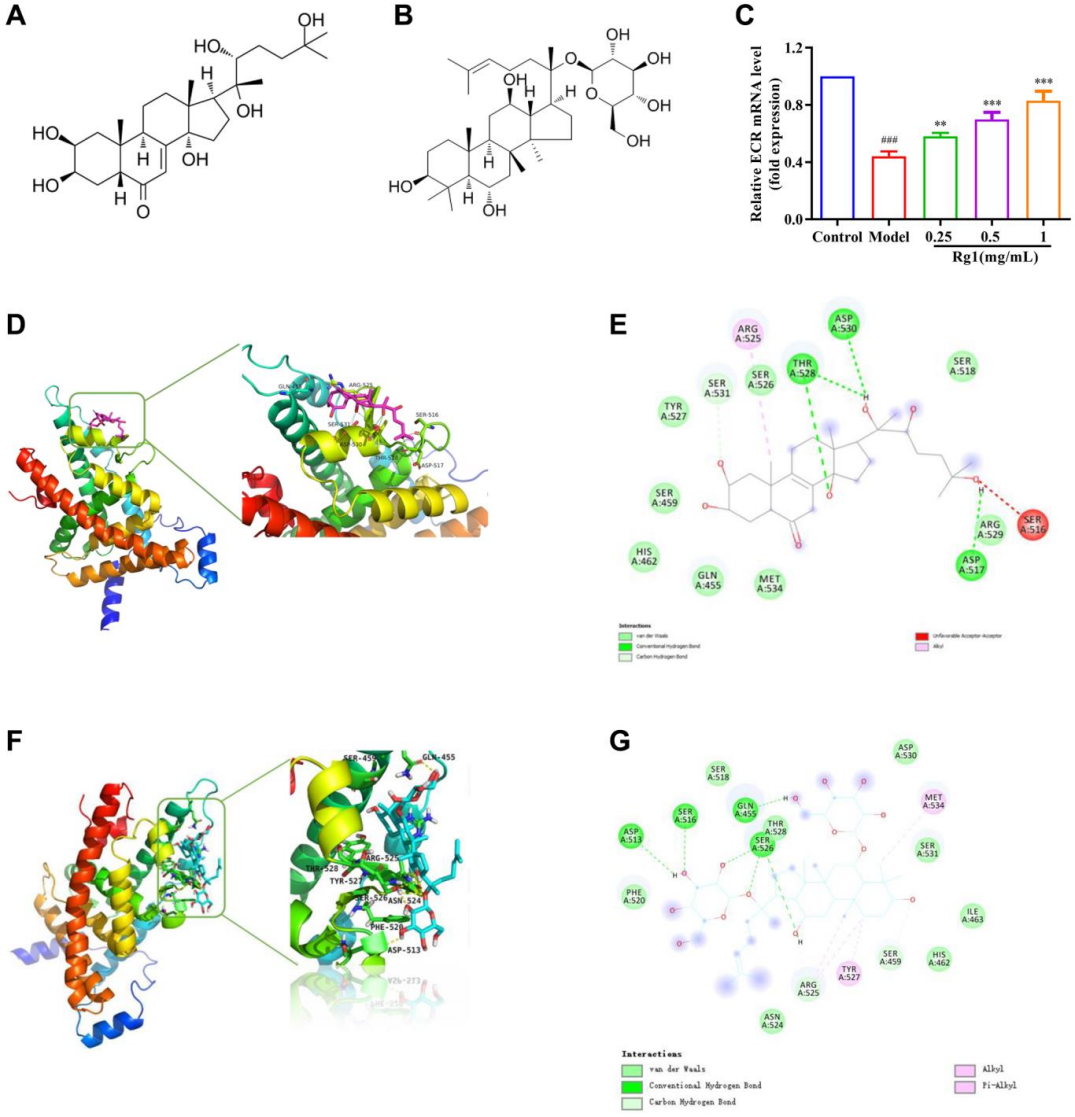
**Effect of ginsenoside Rg1 on ECR.** (**A**) The chemical structure of ecdysterone; (**B**) The chemical structure of ginsenoside Rg1; (**C**) Relative mRNA-level expression of gene encoding ECR; (**D**) 3D diagrams of the docked structure of ecdysterone in the active domain of the ECR; (**E**) 2D diagrams of the docked structure of ecdysterone in the active domain of the ECR; (**F**) 3D diagrams of the docked structure of ginsenoside Rg1 in the active domain of the ECR; (**G**) 2D diagrams of the docked structure of ginsenoside Rg1 in the active domain of the ECR. Results were analysed with one-way ANOVA. Data are shown as the mean ± SD (*n* = 100); ^###^*p* < 0.001 compared with Control (7-day-old female *Drosophila*); ^**^p < 0.01, ^***^p < 0.001 compared with Model (35-day-old female *Drosophila*).

Ginsenoside Rg1 is a steroid compound that is structurally similar to ecdysterone (Figure 4A, 4B). Ecdysterone binds to the hormone receptor ECR on the cell membrane and initiates a transcriptional cascade reaction. Expression level of ECR was decreased in aged *Drosophila* treated with ginsenoside Rg1 was compared to that in young ones and returned to the level in young flies (Figure 4C). ECR is a nuclear receptor (NR) superfamily member with a centrally conserved DNA binding domain (DBD), a non-conserved hinge, a varying N-terminal domain, and a C-terminal ligand binding domain (LBD) [32]. To elucidate the plausible mechanism underlying biological activities, docking simulations were performed. Ecdysterone could bind to the LBD domain of ECR (419-654aa) to form suitable steric complementarity by hydrogen bonding and alkyl interaction with the binding energy of −7.7506 Kcal/mol (Figure 4D, 4E). The results showed that the strongest bonds usually occurred when ginsenoside Rg1 bond to GLN455, ASP513, ARG525, SER526, TYR527 and MET534 in the substrate binding subsite with an affinity energy of −5.9 Kcal/mol. Ginsenoside Rg1 made one significant hydrogen bonded with the LBD domain (Figure 4F, 4G). This interaction was shown to be essential for binding to ECR. Ginsenoside Rg1 could bind to the LBD domain of ECR, which was consistent with the findings from ecdysterone. These results suggested that ginsenoside Rg1 promotes the expression and activity of ECR, which is similar to steroid hormone.

### Ginsenoside Rg1 target ECR to activate the BMP signaling

**Figure 5:**
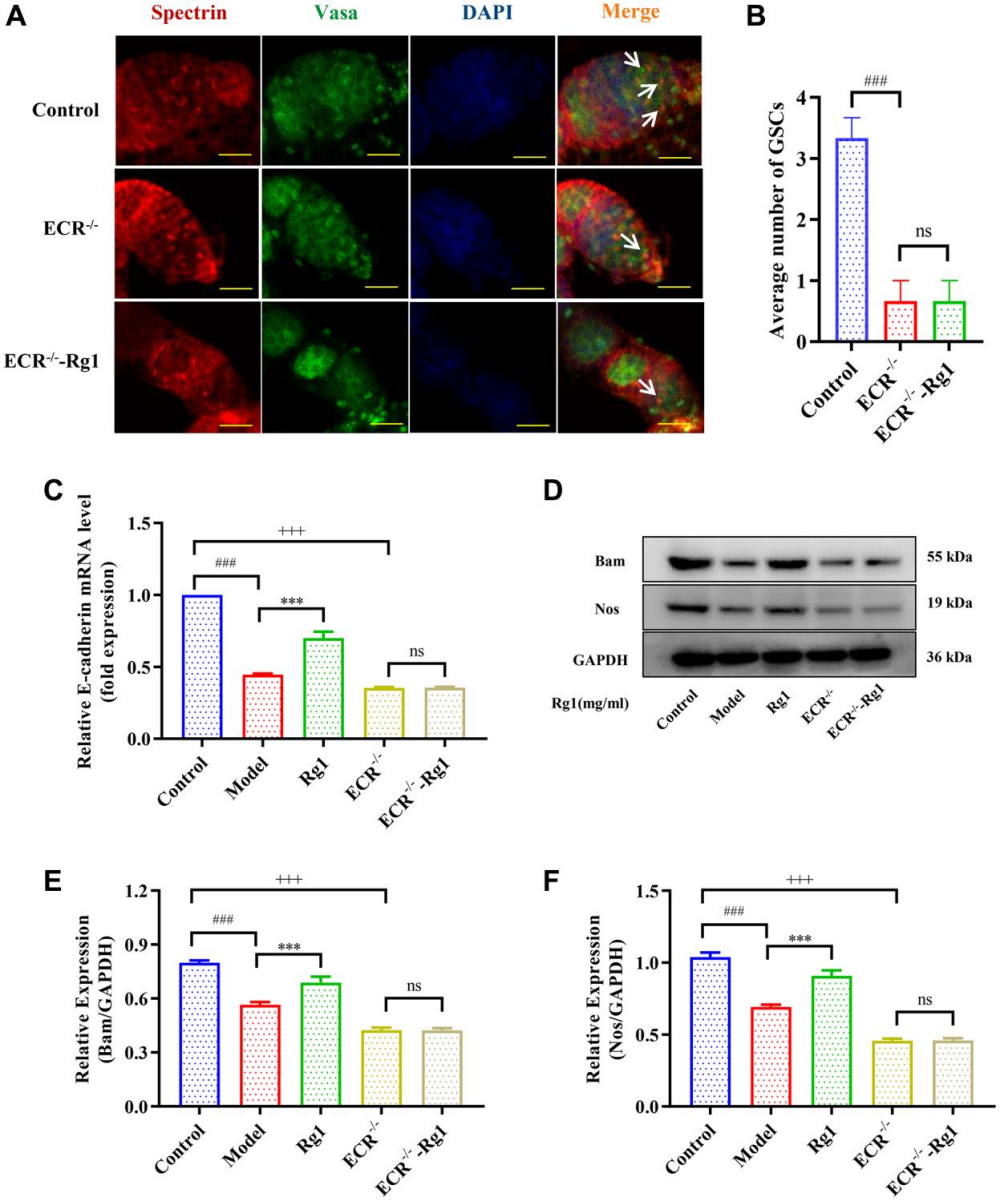
**Effects of ginsenoside Rg1 on GSCs niche in ECR mutant *Drosophila*.** (**A**) GSCs are labeled with α-spectrin antibody (red, fusions), Vasa antibody (green, germ cells), and DAPI (blue, nuclei), Scale bar: 10 μm; (**B**) Average number of GSCs; (**C**) Relative mRNA-level expression of gene E-cadherin (*n* = 100); (**D**) Western blotting analysis of expression of Bam and Nos; (**E**) Relative expression levels of Bam (*n* = 100); (**F**) Relative expression levels of Nos (*n* = 100); GAPDH antibody was used as loading Control. Results were analysed with one-way ANOVA. Data are shown as the mean ± SD (*n* = 100); ^###^*p* < 0.001 compared with Control (7-day-old female *Drosophila*); ^***^p < 0.001 compared with Model (35-day-old female *Drosophila*); ^+++^*p* < 0.001 compared with Control (7-day-old female *Drosophila*); Abbreviation: ns: no significance.

**Figure 6:**
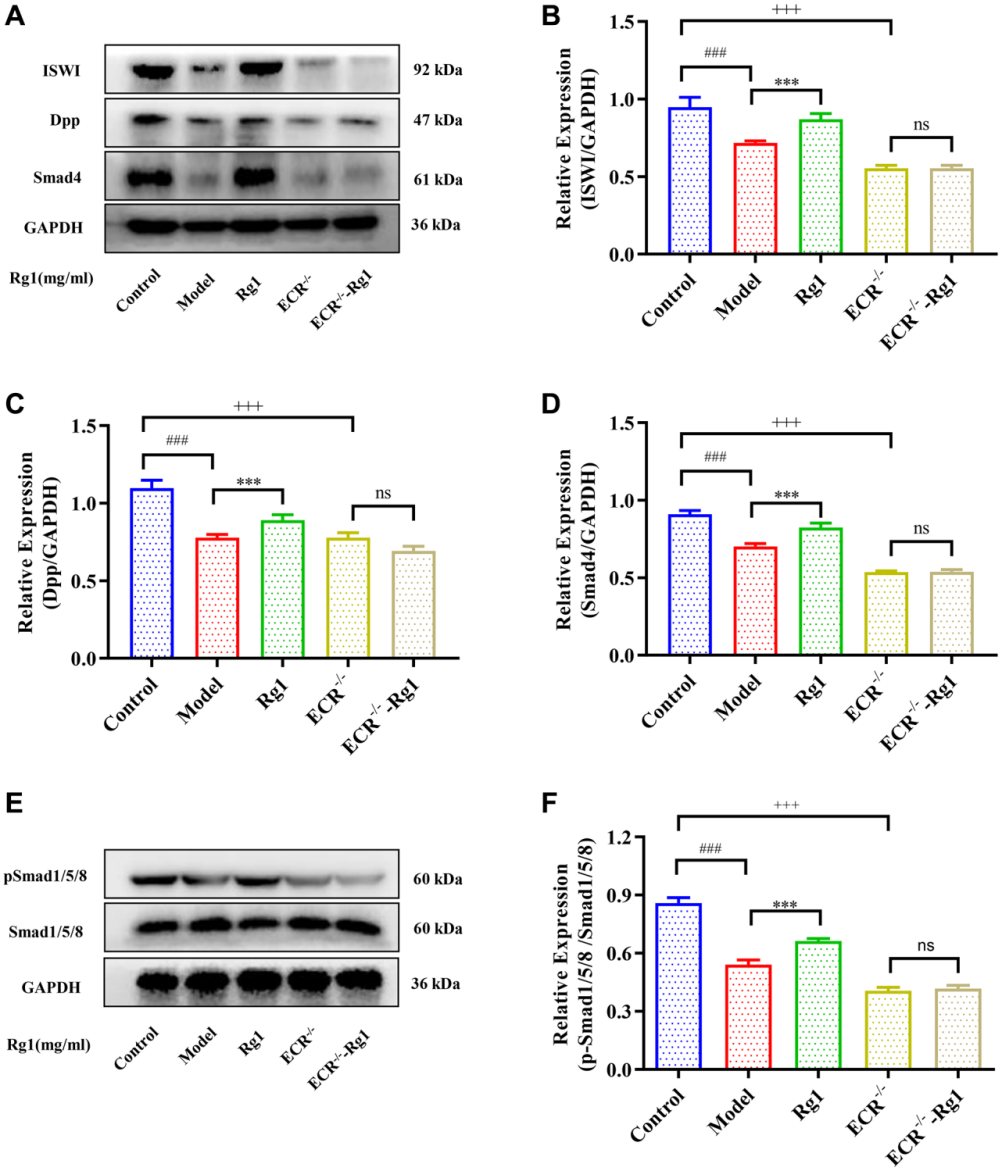
**Effect of ginsenoside Rg1 on niche ECR/BMP signaling in *Drosophila*.** (**A**) Western blotting analysis of expression of ISWI, Decapentaplegic (Dpp) and Smad4; (**B**) Relative expression levels of ISWI; (**C**) Relative expression levels of Dpp; (**D**) Relative expression levels of Smad4; (**E**) Western blotting analysis of expression of pSmad1/5/8; (**F**) Relative pSmad1/5/8 expression levels; GAPDH antibody was used as loading Control. Results were analysed with one-way ANOVA. Data are shown as the mean ± SD (*n* = 100); ^###^*p* < 0.001 compared with Control (7-day-old female *Drosophila*); ^***^p < 0.001 compared with Model (35-day-old female *Drosophila*); ^+++^*p* < 0.001 compared with Control (7-day-old female *Drosophila*); Abbreviation: ns: no significance.

To demonstrate that ginsenoside Rg1 functions similarly to steroid hormones, we investigated the targeting effect of ginsenoside Rg1 on ECR gene knockdown in female *Drosophila*. The average number of GSCs in ECR mutant was 0.67, while the average number of GSCs in young *Drosophila* was 3.33. Ginsenoside Rg1 treatment did not increase the number of GSCs in the ovaries of ECR mutants (Figure 5A, 5B). Compared to wild-type flies, ECR knockout flies produced fewer eggs, pupa, had smaller ovarian tissue, and ecdysterone levels were lower, ginsenoside Rg1 failed to improve these phenomena (Supplementary Figure 3A–3D). Furthermore, the expression of factors that maintain the stemness of GSCs, including E-cadherin, Bam, and Nos, were not increased in ECR mutants by ginsenoside Rg1 treatment (Figure 5B–5F). These results suggested that ginsenoside Rg1 might depend on ECR in maintaining the GSCs stemness in aged female *Drosophila*. The BMP signaling pathway was inhibited when ECR was knocked out. The expression of ISWI, Dpp, Smad4 and pSmad1/5/8 were not restored after ginsenoside Rg1 treatment in ECR mutant *Drosophila*, which was contrary to the findings in aged ones (Figure 6). The above results proved that ginsenoside Rg1 target ECR to activate the niche BMP signaling pathway.

## DISCUSSION

*Panax ginseng* C.A. Meyer has been used as a tonic for thousands of years in China and other Asian countries [33, 34]. It is currently used as a tonic to preserve hormonal homeostasis and fertility in women [35–37]. The pharmaceutical research has shown that ginsenoside can enhance ovarian function and reinstate estrogen secretion [38]. We found that ginsenoside Rg1 played a positive corrective action in the ageing ovaries, including to improve the number and quality of the subgeneration, inhibit ovarian atrophy and restore female hormone levels (Supplementary Figure 2). Previous studies mainly focused on ginsenoside Rg1 improving ovarian function by promoting follicular development and reducing follicular atresia [39]. But follicle regeneration is the key to remodeling ovarian reserve function. Whether ginsenosides Rg1 can promote follicle regeneration has not been reported.

Follicular regeneration depended on the regeneration potential of GSCs, which was regulated by their niche [40]. Therefore, improving the GSCs niche could increase follicle reserve, which provided a new insight for delaying ovarian aging. So far, the research on stem cell niche is still in its infancy. It is extremely difficult to identify a GSCs niche in mammals, but relatively easy in the model animal *Drosophila*, whose gonads have been applied as a visualization system for the GSCs niche. The GSCs number was reduced, and the GSCs niche was damaged in older female *Drosophila*, which was consistent with previously reported results [41]. Ginsenoside Rg1 inhibited the age-induced decrease of stem cell-related factors Bam, Nos and E-cadherin, and increased the number of GSCs. These findings indicated that ginsenoside Rg1 could enhance stem cell self-renewal through avoiding age-induced niche imbalance.

**Figure 7:**
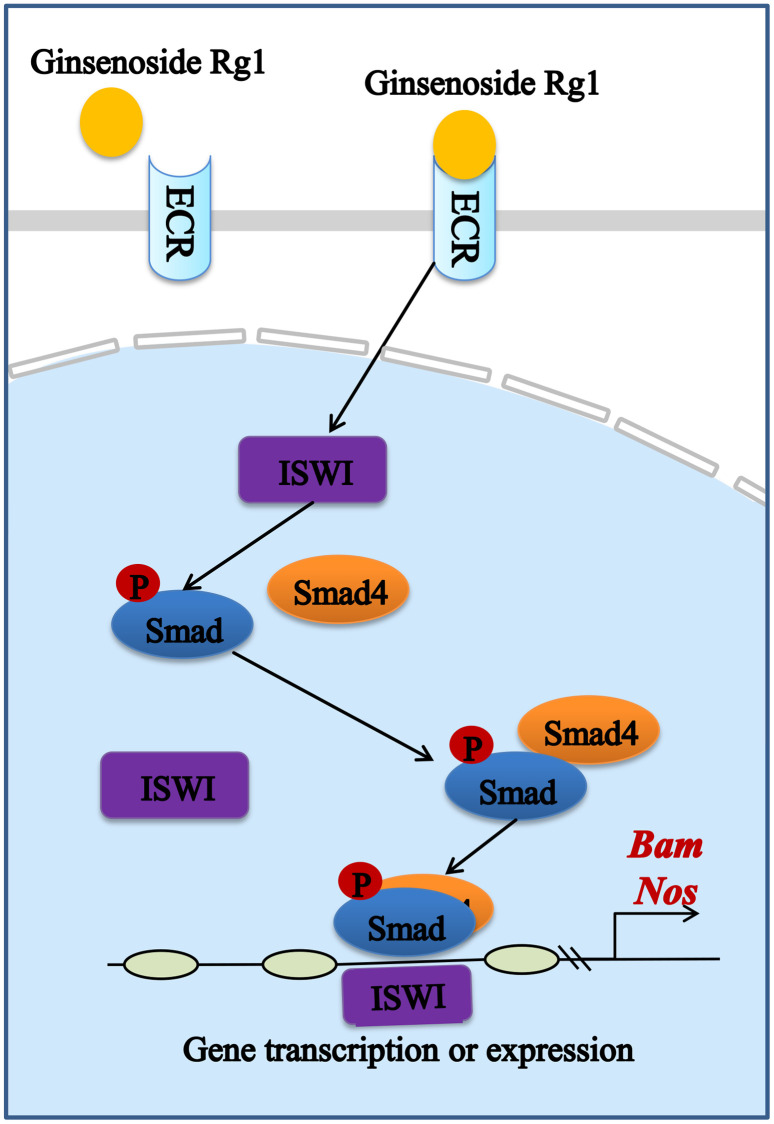
Schematic diagram of the signaling pathway of the mitigating effect of ginsenoside Rg1 improves the niche to increase GSC number by targeting ECR.

Estrogen is produced by the ovaries and is responsible for regulating the stability of the GSCs niche. Studies have demonstrated that estrogen bound to nuclear receptors in mammals can trigger conformational changes of the receptors to allow transcriptional coactivator binding and transcription initiation complex assembly, playing an important regulatory role in the GSCs niche [42, 43]. Similar to mammals, ecdysterone attaches to the steroid hormone receptor ECR in female *Drosophila*, activating the production of early E74 and Br transcription factors to regulate niche balance (Supplementary Figure 3) [44]. We also obtained the same results (Supplementary Figure 4). It is further proved that the ginsenoside Rg1 had a complementary replacement for the lack of ecdysterone in the aging ovaries, which could bind effectively to the receptor ECR. In addition, ECR can be involved in BMP signaling through the activation of chromatin remodeling factor ISWI [45]. The BMP signaling in niche is a central signal that regulates the GSCs stemness. GSCs in the niche secrete BMP ligands that promote the phosphorylation of Samd1/5/8 [46]. pSmad1/5/8 forms a complex with Smad4 to activate the promoter activity of stem cell maintenance factor Nos and differentiation factor Bam to increase the number of GSCs [47, 48]. In this study, ginsenoside Rg1 activates ECR to increase BMP signal strength (Figure 7). In addition, BMP signal intensity as well as GSCs number were not recovered in ECR mutant female *Drosophila* treated with ginsenoside Rg1. This suggested that ginsenoside Rg1 regulates the niche to maintain GSCs self-renewal through targeting ECR/BMP signaling pathway.

It was found that ginsenoside Rg1 through targeting ECR/BMP signaling pathway to inhibit age-induced GSCs loss. It is noteworthy that ginsenoside Rg1 acts as a hormone-like role to compensate for age-induced loss of endogenous estrogens, which was beneficial for women with needs of prolonged childbearing potentials and delayed ovarian aging. Further studies would be needed to explore the role and mechanism of ginsenoside Rg1 to prevent and treat estrogen-induced diseases. This may provide a new solution to the disease associated with ovarian aging and menopausal syndrome.

## Supplementary Materials

Supplementary Figures
