# Supplementary material for: New insights into ginsenoside Rg1 regulating the niche to inhibit age-induced germline stem cells depletion through targeting ECR/BMP signaling pathway in Drosophila
Source: Aging (Albany NY). 2024 Feb 14;16(4):3612–30. doi: 10.18632/aging.205548 (PMC10929810; doi:10.18632/aging.205548)
Supplement: Supplementary Figures [file aging-16-205548-s001.pdf]

SUPPLEMENTARY FIGURES

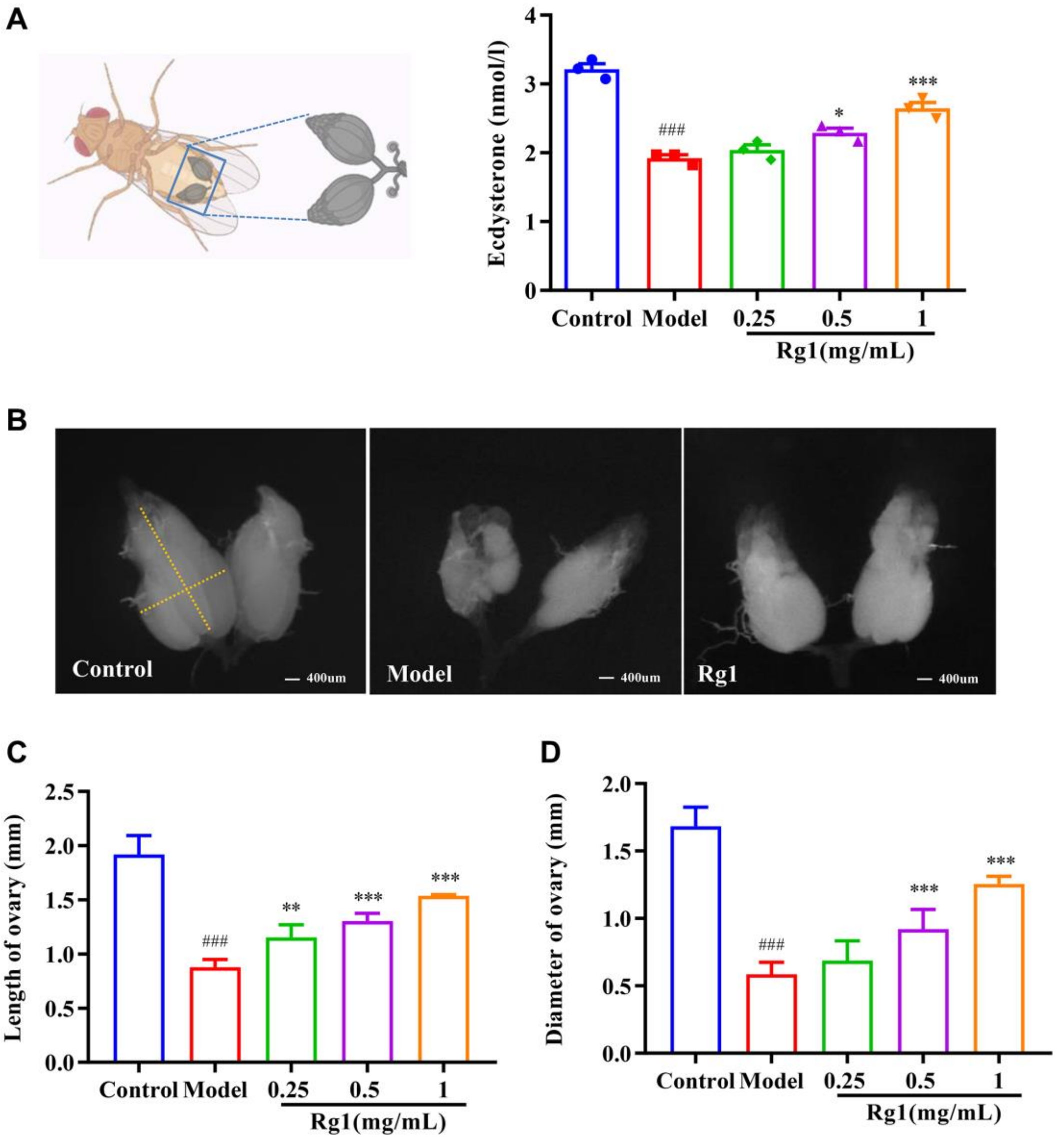

Supplementary Figure 1. Effects of ginsenoside Rg1 on ovarian function and aging in *Drosophila*. (**A**) Ecdysterone level; (**B**) Ovary size (n = 10), Scale bar = 400 µm; (**C**) Length of ovary; (**D**) Diameter of ovary; Results were analyzed with one-way ANOVA. Data are shown as the mean ± SD; $^{###}p < 0.001$ compared with Control (7-day-old female *Drosophila*); $^{*}p < 0.05$, $^{**}p < 0.01$, $^{***}p < 0.001$ compared with Model (35-day-old female *Drosophila*).

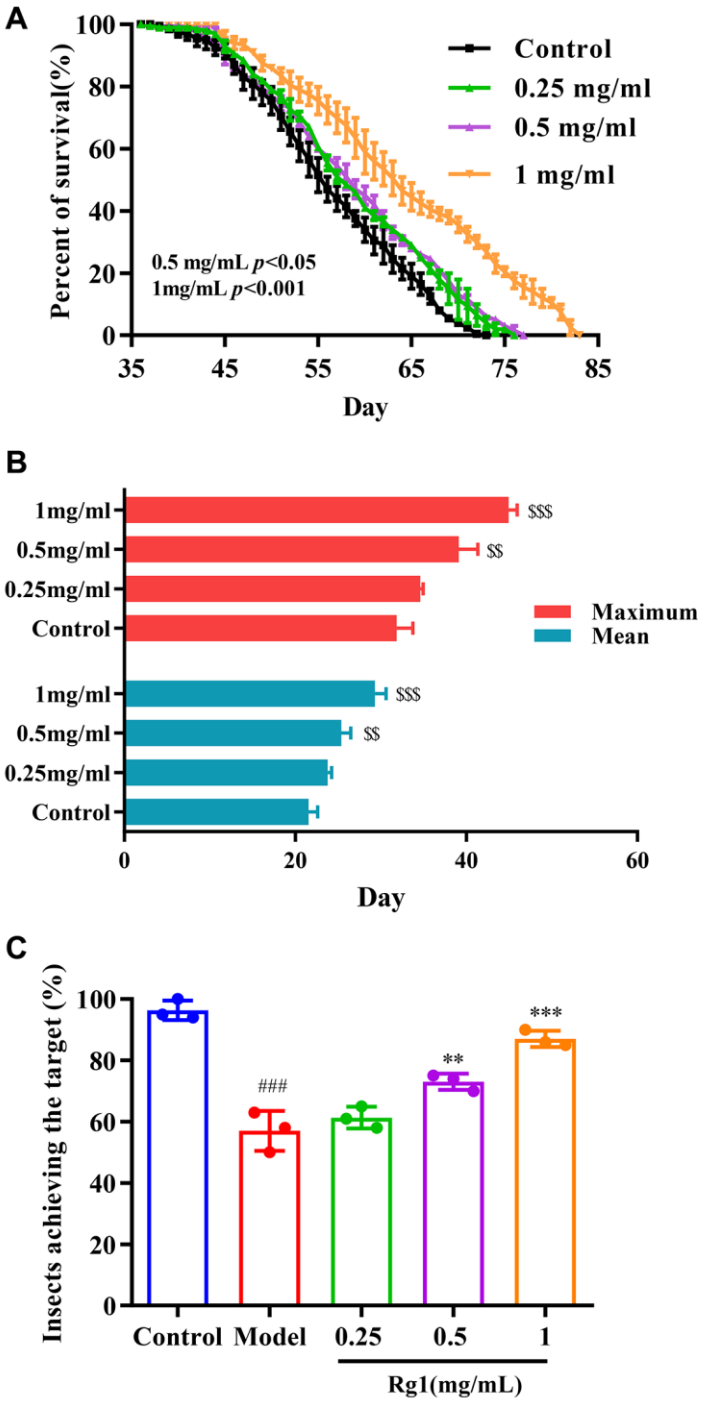

Supplementary Figure 2. Effects of ginsenoside Rg1 on viability in *Drosophila*. (**A**) Percent of survival (n = 200); (**B**) Mean and maximum lifespans; (**C**) Climbing ability (n = 100); Results were analyzed with one-way ANOVA. Data are shown as the mean ± SD; $^{###}p < 0.001$ compared with Control (7-day-old female *Drosophila*); $^{*}p < 0.05$, $^{**}p < 0.01$, $^{***}p < 0.001$ compared with Model (35-day-old female *Drosophila*); $^{\$\$}p < 0.01$, $^{\$\$\$}p < 0.001$ compared with Control (35-day-old female *Drosophila*).

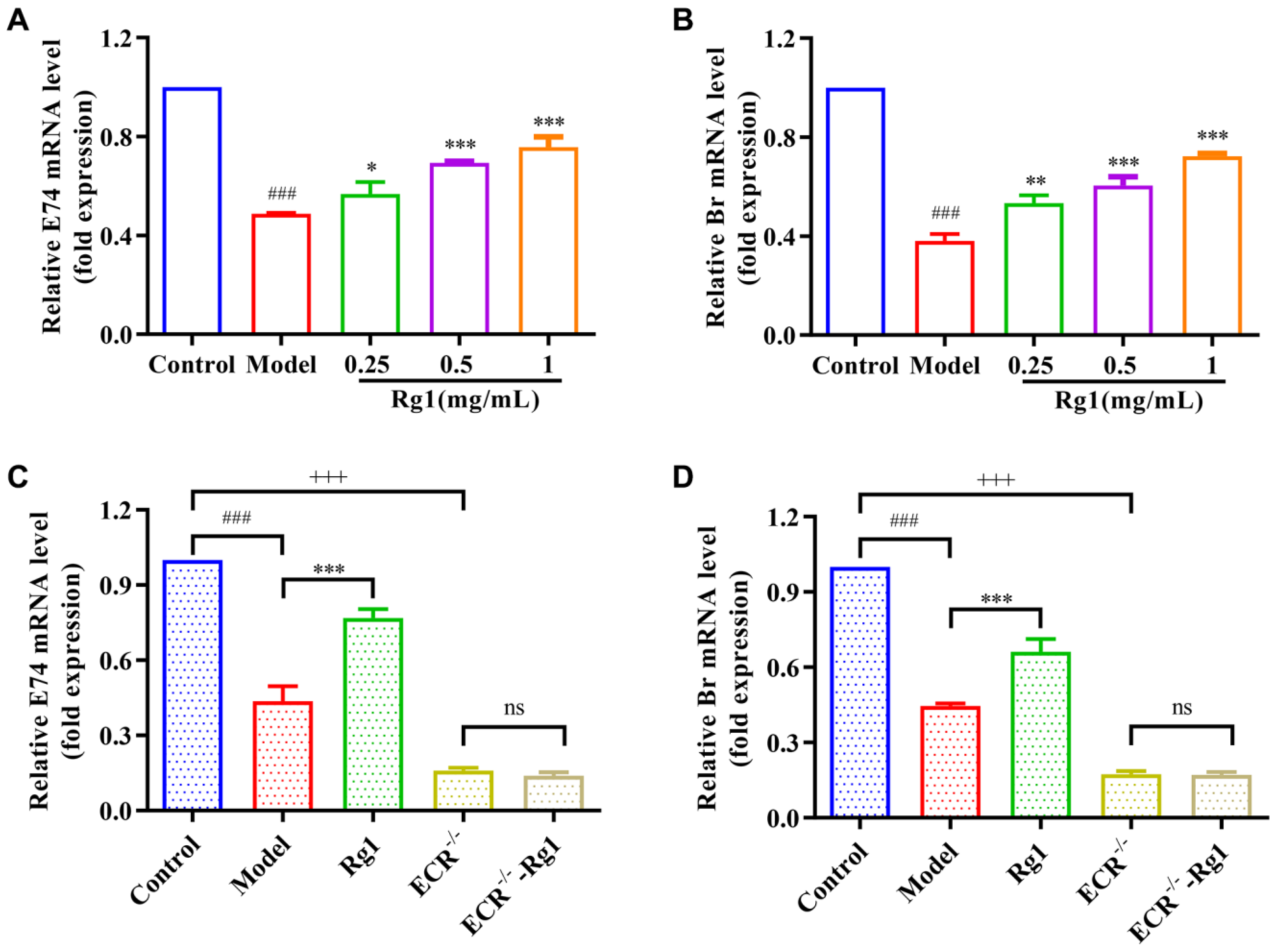

Supplementary Figure 3. Effect of ginsenoside Rg1 on downstream signal of ECR. (**A**) Relative mRNA-level expression of gene encoding inducible protein E74 in elderly female *Drosophila*; (**B**) Relative mRNA-level expression of gene encoding transcription factor Br in elderly female *Drosophila*; (**C**) Relative mRNA-level expression of gene encoding inducible protein E74 in ECR mutant *Drosophila*; (**D**) Relative mRNA-level expression of gene encoding transcription factor Br in ECR mutant *Drosophila*. Results were analysed with one-way ANOVA. Data are shown as the mean ± SD (n = 100); $^{###}p < 0.001$ compared with Control (7-day-old female *Drosophila*); $^{*}p < 0.05$, $^{**}p < 0.01$, $^{***}p < 0.001$ compared with Model (35-day-old female *Drosophila*); $^{+++}p < 0.001$ compared with Control (7-day-old female *Drosophila*); Abbreviation: ns: no significance.

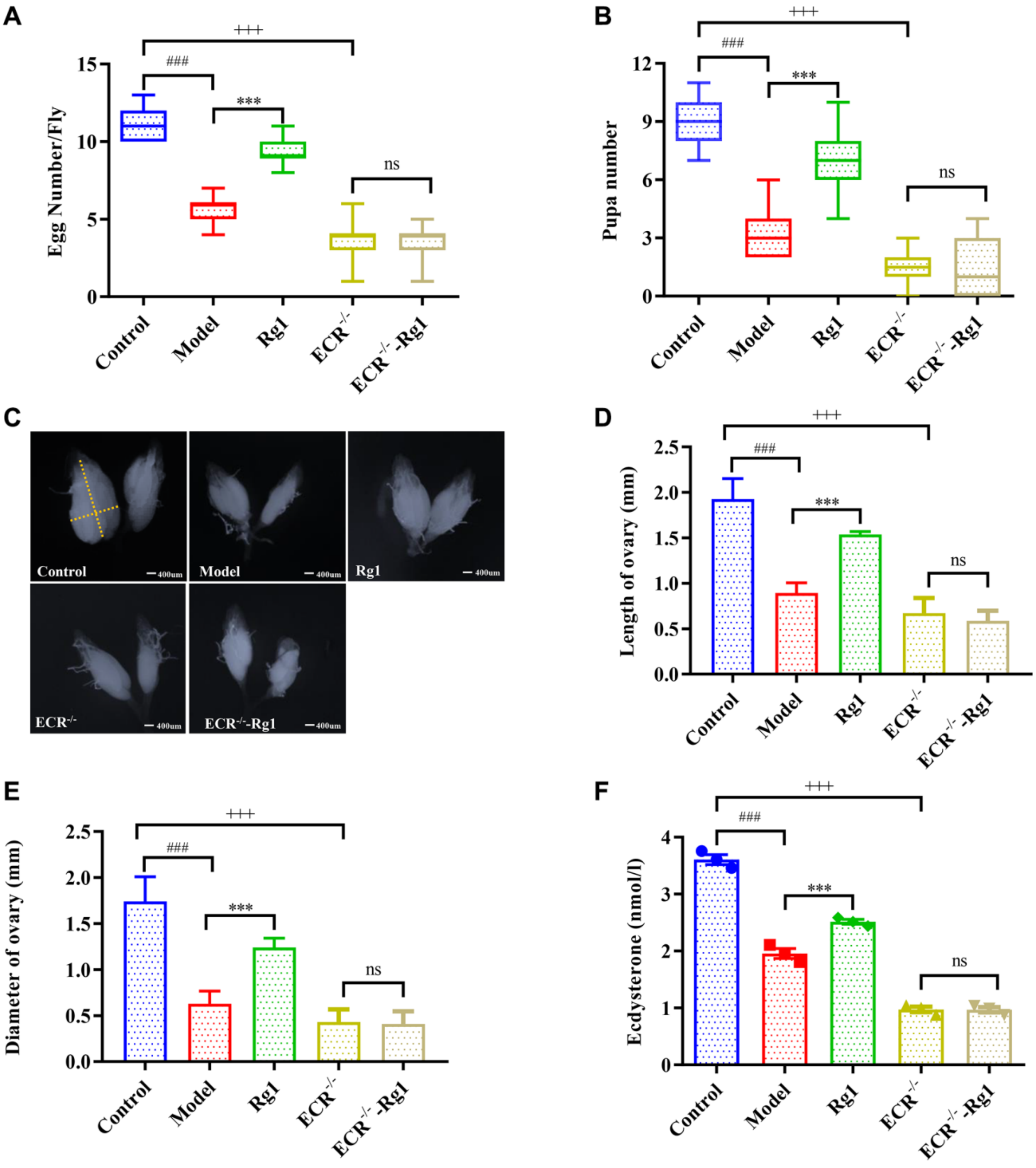

Supplementary Figure 4. Effects of ginsenoside Rg1 on ovarian function in ECR mutant *Drosophila*. (**A**) Number of eggs (n = 100); (**B**) Number of pupae (n = 100); (**C**) Ovary size (n = 10), Scale bar = 400 um; (**D**) Length of ovary; (**E**) Diameter of ovary; (**F**) Ecdysterone level (n = 100). Results were analysed with one-way ANOVA. Data are shown as the mean ± SD (n = 100); $^{###}p < 0.001$ compared with Control (7-day-old female *Drosophila*); $^{***}p < 0.001$ compared with Model (35-day-old female *Drosophila*); $^{+++}p < 0.001$ compared with Control (7-day-old female *Drosophila*); Abbreviation: ns: no significance.
